# Moral distance, AI, and the ethics of care

**DOI:** 10.1007/s00146-023-01642-z

**Published:** 2023-03-23

**Authors:** Carolina Villegas-Galaviz, Kirsten Martin

**Affiliations:** 1 Technology Ethics Center, University of Notre Dame, 204 O’Shaughnessy Hall, Notre Dame, IN 46556 USA; 2 IT, Analytics, and Operations, University of Notre Dame, South Bend, IN USA

**Keywords:** Artificial intelligence, AI, Moral distance, Ethics of care, AI ethics

## Abstract

This paper investigates how the introduction of AI to decision making increases moral distance and recommends the ethics of care to augment the ethical examination of AI decision making. With AI decision making, face-to-face interactions are minimized, and decisions are part of a more opaque process that humans do not always understand. Within decision-making research, the concept of moral distance is used to explain why individuals behave unethically towards those who are not seen. Moral distance abstracts those who are impacted by the decision and leads to less ethical decisions. The goal of this paper is to identify and analyze the moral distance created by AI through both proximity distance (in space, time, and culture) and bureaucratic distance (derived from hierarchy, complex processes, and principlism). We then propose the ethics of care as a moral framework to analyze the moral implications of AI. The ethics of care brings to the forefront circumstances and context, interdependence, and vulnerability in analyzing algorithmic decision making.

## Introduction

At close range the resistance to killing an opponent is tremendous. When one looks an opponent in the eye, and knows that he is young or old, scared, or angry, it is not possible to deny that the individual about to be killed is much like oneself. (Grossman 1995)

When talking about remote fighting and drones, the issue of moral distancing means the disappearance of the vulnerable face of the opponent, which apparently, makes it easier to kill (Coeckelbergh 2013; see also Cummings 2004). The distance created by technology can block the empathy that would arise when seeing the face of the *opponent*. This means both developers and those using AI lack the ability to identify or understand the concerns and mental states of subjects impacted by AI (Montemayor et al. 2021). However, it does not exempt one from moral responsibilities. When deciding to give or not a loan or a mortgage, or to deny or grant an insurance premium, it could be easier to deny specific opportunities if, thanks to the use of emerging technologies, such as artificial intelligence (AI), we do not see the vulnerabilities and specific characteristics of people. Is easier to *kill*. For example, is Amazon firing delivery drivers easier when the assessment and task are fully automated with AI? If so, what framework could help firms better see the impacts of those morally relevant decisions in design and use?

With AI we refer to algorithms that “sift through data sets to identify trends and make predictions” (Martin 2019b, p 836). If the use of AI impacts moral distancing—where decision reduces to data, ignores circumstances, vulnerabilities, and the specific harm that can be done to that individual—the firm misses the moral implications of the decisions for which is responsible. The firm is blinded to the impact of its decisions behind the veil of AI while also being responsible for those decisions.

Companies continue to repeat the same mistakes. While bias in facial recognition programs has been known for years (Buolamwini and Gebru 2018), Google and Facebook, for example, have struggled with issues related to AI and race, when their facial recognition algorithms labeled Black individuals as “primates” (Facebook in 2021). Companies apologized, but mistakes persist and may continue until dealing with them become a priority for their leaders (Mac 2021).

The purpose of this paper is to identify the moral distance that can be created in the development and deployment of AI. While other technologies, such as the drones mentioned above, can exacerbate moral distance, we conceptualize that the use of AI can contribute to moral distancing in two ways. First, with the elimination of face-to-face interactions (creating a distance of space, time, and culture), the use of AI creates *proximity distance*. Second, the use of AI creates what we call *bureaucratic distance* derived from hierarchy, complex processes, and principlism. The essential point is that “the very distance between an act and its ethical consequences (ethical distance) may also play a determining role—if not always in the same way—in the transition process” (Zyglidopoulos and Fleming 2008). While distancing in decision-making can be useful in bringing in a degree of impartiality and standardizing processes and procedures, the idea behind moral distancing is that such distancing can have downsides. We wish to address these downsides in terms of AI. AI can be better designed and deployed when the downsides of moral distancing are addressed.

To help ameliorate the downsides of moral distancing from the use of AI, we propose the theory of ethics of care (Gilligan 1982; Noddings 1984) as a moral framework to analyze technology and AI's moral implications. The notion of care within ethics promotes reference to vulnerabilities, context, and empathy in ethical decision-making (Gilligan 1982; Noddings 1984; see also French and Weis 2000; Held 2006). Within technology ethics, ethics of care may be the way to foreground notions of culture, diversity, and the “other” (Hersh 2016).

We argue that the ethics of care addresses the issue of moral distancing since the theory “associates moral action with meeting the needs, fostering the capabilities, and alleviating the pain and suffering of individuals in attentive, responsive, and respectful ways” (Engster 2011). However, the ethics of care does not imply deference to feelings of pity or altruism, but rather to take ethical reasoning beyond the reduction to principles and the consideration of purely quantifiable variables, and to consider different points of view (hear different voices), interdependent relationships, context and circumstances, and individual vulnerabilities. The ethics of care is proposed as a complementary and integrative proposal to augment existing work in AI Ethics.

The paper structure is as follows. First, we conceptualize the problem of moral distance and explain how AI and technology exacerbate the issue. In the second part, we present the ethics of care and emphasize four ideas within the ethics of care, there we explain how each could help to address the downsides of moral distancing with AI.

## Moral distance and AI

In his pioneering study, *Modernity and the Holocaust*, Zygmunt Bauman argues that some “moral sleeping pills,” such as bureaucracy, may blur ethical concerns for those who are far from us, creating the problem of moral distance. Indeed, in some circumstances, distance may favor impartiality, or limit the possibilities of favoritism or influences of power. However, the issue of distancing in morality appears when “the natural invisibility of causal connections in a complex system of interaction, and the `distancing' of the unsightly or morally repelling outcomes of action” arise to the point of “making invisible the very humanity of the victims” (Bauman 1989). When developing his argument, Bauman takes the Milgram experiment, where the psychologist studied how obedience to authority could turn the participant to perform acts against their conscience and do great harm to another person.

Ethical implications of moral distance have been the center of studies focused on business decisions (Huber and Munro 2014; Mellema 2003; Zyglidopoulos and Fleming 2008; see also Jones et al. 2005). In the context of complex situations, such as organizational corruption in cases such as Enron, Arthur Andersen, and WorldCom, the concept of moral distance explains how individuals performed unethical acts even in cases when they claim to have principles and values against those kinds of acts (see Mellema 2003; also Zyglidopoulos and Fleming 2008). There, the problem of moral distance is related to the conceptions of limits, is about boundaries and how they “demarcate not only physical, political, and other space but the moral space of inclusion and exclusion determining the limit and extent of our moral concern” (Chatterjee 2003). In that sense moral distance differs from moral disengagement, because in the former there is an actual distance that limits the whole comprehension of the moral context, whereas the latter refers to the belief of people who convince themselves that they are causing no harm or acting wrong because ethical principles do not apply to them (Bandura 2002).

Mark Coeckelbergh (2013) was one of the first to address the relationship between distance, morality, and technology. In his work, Coeckelbergh takes the practice of using drones in remote fighting to illustrate “the claim that new technological practices that aim to bridge physical distance create more moral distance and make it difficult for people to exercise moral responsibility” (p. 88). Previously, Bauman (1989) also talked about the role of technology in increasing and exacerbating the problem of distance and morality when he explains that the issue “becomes particularly acute in our modern, rationalized, industrial technologically proficient society because in such a society human action can be effective at a distance, and at a distance constantly growing with the progress of science, technology, and bureaucracy.”

Moral distance has two components. The first is proximity, in space, time, and culture, where individuals tend to behave ethically regarding those in close proximity to them. The second component is related to bureaucracy and hierarchy; when a person's act is a small part of an extensive process, a kind of moral diffusion of responsibility appears, as in the problem of ‘many hands,’ leading to moral distancing. Moral distance could be created by the temporal, physical, and cultural distance of a person between his or her act and its consequences, and it can be the result of an organization’s bureaucracy (Huber and Munro 2014; Zyglidopoulos and Fleming 2008).

Distance in decision-making has virtues. Decision-makers may be less prone to favoritism and better able to have standards or policies. Distancing is useful for a better degree of impartiality. Our goal here is to identify the potential downsides of moral distancing with AI to offer a framework to help ameliorate these risks. We extend the examination of technology implications on moral distance addressing how AI exacerbates the problem.

Table 1 is a summary of the types of moral distance and of how AI exacerbates the problem. This table also contains an explanation of how we develop in our paper the specific way in which AI creates moral distance. AI exacerbates moral distance in a very concrete way because there is no human in the loop.

**Table 1:** Outline of the types of moral distance and the explanation of how AI exacerbates the problem

Moral distance	Subtypes and definition		With AI
Proximity distance	Physical	No face-to-face interactions	Decision-making without seeing/interacting with those affected by it
	Temporal	How own actions impact the future (and future generations)	How models can change with the introduction of new data
	Cultural	Using the same value to judge different cultures	AI systems are designed in a culture/society and deployed in a different one Framing subjects from different cultures using the same algorithm
Bureucratic distance	Hierarchy	The power over subordinates	Influenced power of companies over developers Deference of decisions to AI as an authority
	Complex Processes	The problem of many hands and blurred responsibility	Who is responsible for AI decision-making consequences? Algorithmic inscrutability
	Principlism	Reduction of morality to principles or a blind attachment to moral guides	How principles alone have a limited impact on AI ethics

### Proximity distance

According to Bauman (1989), there is an inverse ratio of readiness to cruelty and proximity to victims, “it is difficult to harm a person we touch. It is somewhat easier to afflict pain upon a person we only see at a distance. It is still easier in the case of a person we only hear. It is quite easy to be cruel towards a person we neither see nor hear.” Moreover, many ethicists such as Aristotle (in *Rethoric*) or Hume (in *A treatise of human nature*) have talked about the problem of proximity in morality and how distance affects moral action (Chatterjee 2003). “Ethical traditions that base morality on human nature claim that distance over time and place matters morally because humans are by nature unsuited to show equal concern to distant people and events compared to those near in time and place” (Chatterjee 2003, p. 327). For example, Bezzubova (2020) makes a convincing argument that the use of virtual reality introduces derealization and depersonalization in how we perceive ourselves through the introduction of distance to ourselves. However, distance does not exempt from moral responsibilities.

Regarding proximity distance, there might be a physical, temporal, and cultural impact in distancing.

#### Physical distance

Regarding *physical distance* affecting morality, Bauman (1989) argues that moral inhibitions are tied to human physical proximity, hence moral inhibitions may not act at a distance. For example, Bauman says that “the increase in the physical and/or psychic distance between the act and its consequences …quashes the moral significance of the act and thereby pre-empts all conflict between the personal standard of moral decency and immorality of the social consequences of the act.” This implies that the physical distance allows the abstraction of the acting subjects, annulling (for them) the moral meaning of the act, which may be the cause of them to act against their principles.

In the field of technology, the physical distance created by information technology is described by Coeckelbergh's (2013) explanation of moral distance in pilots’ remote fighting with drones. In remote fighting, it is easier for soldiers to kill; in body-to-body fighting, soldiers comprehend the opponent as a similar person, as an equal. The close contact opens the possibility of feeling empathy, which impacts the soldiers’ decisions to kill. The author presents moral distance as a moral-epistemological problem since those using the specific technology (due to the distance facilitated by the tool) do not fully know the possible outcomes of their action. Bolstered on the work of Heidegger and Levinas regarding distance, technology, and morality, Coeckelbergh states that technology creates a new world for those using it: “The technology and the distance it creates does not only produce a barrier between our empathic capacity and the opponent, it changes the very way we perceive that opponent” (p. 93). Within the same argument, scholars have defended that technology has ethical implications because it limits engagement and commitment (Borgmann 1987), and is a tool to eliminate personal vulnerabilities (Dreyfus 2008).

Within AI the idea that algorithms can increase anonymity and psychological distance has been identified as a relevant threat in the possible malicious use of AI (Brundage et al. 2018). According to Brundage et al. (2018), many tasks involve communicating face-to-face, and “by allowing such tasks to be automated, AI systems can allow the actors who would otherwise be performing the tasks to retain their anonymity and experience a greater degree of psychological distance from the people they impact.” This means that proximity distance creates an epistemological or psychological distance with moral implications. Also, Coeckelbergh (2015) states that AI and automation alienate individuals from material reality since “we now work in ways that no longer require intense and direct engagement with that material reality … it thereby creates a gap, a distance, between us and nature.” Specifically, in the healthcare domain, Montemayor et al., note the difficulty AI would have in enacting empathy in the care of others (2021).

For example, Amazon algorithmically rates its drivers and automatically fires them by email. When a human is asked to assess the ability of a driver, they may have been a driver themselves, understand what the data means in the context of a given route, and would be required to interact with the individual before telling them they are fired. However, if AI is the boss who fires the employee, the program does not know that the firm is firing an Army veteran who claims to have done nothing wrong in his job (Soper 2021) or a mother affected by the economic crisis of the COVID-19 pandemic to whom before being fired was told (from the same Amazon app) that she was doing a "great" job, in a scale of Fantastic, Great, Fair or At Risk (Gilbert 2021). If the whole process, from rating to firing, is automated, the company is ill-equipped to address specific circumstances affecting the data.

#### Temporal distance

Zyglidopoulos and Fleming (2008) describe *temporal distance* as the type that “refers to how far into the future the consequences of one’s acts are. The further ahead in time these consequences are, the easier it will be for individuals to discount the moral consequences of their act.” With this concept, the authors explain how the short-termism of business influences temporal distance. With a focus on immediate results, people lose sight of the future consequences of their actions. Also, Stephen M. Gardiner (2003) talked about temporal moral distance in line with what the author calls *intergenerational* ethics, or the obligations of one generation to future people. The said means that there is a problem of moral distance when a person's acts affect (even without being fully conscious) future generations.

Within the discussion regarding the ethics of technology and time, the philosopher Hans Jonas (see Jonas 1984) is one of the prominent voices. According to Jonas, technology enlarges the impact of human action, and with what humans do here and now, thinking of their own goals, massively influences the lives of millions of people in other places and in the future. Furthermore, those affected have no voice or vote in this regard (Jonas 1984).

Within AI the problem of this type of distancing appears in the temporal distance between the development of AI models and their deployment. Also, it appears in how models can change with the introduction of new data. Since AI is a socio-technical system it can be essentially impacted by changes in context, sometimes out of the hands of those who design, develop, and deploy AI. The problem of time and moral distance focuses on unintended and unforeseen consequences and how these are difficult to predict, both short and long-term.

An example of this subtype of distance could be the AI Microsoft’s chatter bot Tay, released on Twitter in 2016. Since the bot learned to reply based on interaction, some hours after its introduction to the platform, it began to use offensive language. To consider this example one should set aside the possible negligence of the designer in not considering the popular culture of teenagers. Here the focus is on how algorithms change over time with the introduction of new data.

#### Cultural distance

Nicholas Rescher (2003) explains the moral significance of cultural distance when talking about judging with external moral standards. The idea refers to how morality and ethical standards should not be separated from the culture where they emerge. Principles and values are bolstered in a historical-cultural context, and one should not judge remote people by the standards of different societies and cultures. According to Rescher, that does not entail “an indifferentist relativism, but rather the contextualism of a situationally determinate value system” (p. 477).

AI exacerbates the cultural distance issue by automating ethical decision-making and the fact that the same model may be deployed in different cultures. The said means that, the problem of AI and cultural distance appears when AI systems that are designed by a certain individual or group in a specific culture or society migrate to be deployed in a different culture. These models, developed from culturally-myopic training data, encode the relative importance of rules and principles that will be used to make decisions in the future. If principles and values are rooted in society, to not fall into a problem of moral distance, developers should consider the moral specificities of each culture. Many cultural factors contribute to moral judgments such as religion, demographics (like population density or economics), the history of the own culture, and the like (Graham et al. 2016). Scholars have argued for the need to engage AI ethics with diverse cultures (Segun 2021).

Nissenbaum makes a similar call for understanding the appropriate flow of data as defined within context. To respect privacy expectations and norms, one must first understand the context in which the data was shared or gathered to then identify the appropriate flow of the data with who has access to the data as well as how the data will be used (Nissenbaum 2009).

The example of *learning analytics*, which refers to the measurement, collection, and analysis of student data in higher education (Slade and Prinsloo 2013, Prinsloo and Slade 2017), illustrates this type of distance. With the knowledge that algorithmic decision-making has the power to shape social life, scholars in the field of ethics and *learning analytics*, argue that there are tensions in research on using algorithms to decide things like who gets accepted into institutions or who can access student funding (Prinsloo 2020). Universities have always used quantifiable variables, like GPA, but AI exacerbates the moral distance that the blindness attachment to those variables can create.

A university would design an acceptance program that must accommodate applicants from different cultures. However, grades have different meanings in different countries. In the case of Spain, only one in twenty students obtain the highest grade, so the scale conversion to a country with a different system (in which more than one student can obtain the highest grade) rates a Spanish candidate lower. Also, the relevance that different variables have on a student's performance varies according to culture. Some cultures value extracurricular activities, volunteering, or networking, and for some others, these may not be significant. If an algorithm automatically decides without considering student nationalities, international students will lose a place they merit. Moreover, if a model frames applicants from the same culture or country as inappropriate, students from disadvantaged neighborhoods or countries may not be the right fit for acceptance according to some models. However, automating that decision could have a great impact in the future with the marginalization of those areas, or entire societies, leaving them with no possibility of prospering.

### Bureaucratic distance

The second form of moral distance is bureaucratic distance. For Bauman (1989), bureaucracy functions as a moral sedative since it is programmed to seek the optimal solution and “to measure the optimum in such terms as would not distinguish between one human object and another, or between human and inhuman objects. What matters is the efficiency and lowering of costs of their processing” (p. 104). Hence, bureaucracy is about procedures of formal rationality, with hierarchies, and complex processes where one's actions are a small part of a bigger objective and should reduce to follow scripts.

Those scripts abstract “the real consequences of the defects … into a set of depersonalized figures and formulae” (Zyglidopoulos and Fleming 2008). The key idea is that proximity distance and bureaucratic distance contribute to creating an "inhuman context" (Huber and Munro 2014) that depersonalized those individuals who will be affected by the consequences of the decision. Within the bureaucratic moral distance, here we explain three subtypes of distancing: hierarchy, complex process or the problem of ‘many hands,’ and principlism.

#### Hierarchy

Hierarchy increases moral distance because individuals tend to act against their principles when an authority demands (as in Milgram’s experiment). Bauman (1989) identified the problem of hierarchy and bureaucracy as one of the main drivers of moral distance. Bauman says that in a bureaucracy what matters is “how smartly and effectively the actor fulfills whatever he has been told to fulfill by his superiors, [which] in addition to giving orders and punishing for insubordination, they also pass moral judgements—the only moral judgements that count for the individual's self-appreciation” (Bauman 1989). Individuals tend to hand over their responsibility for their actions to those who have ordered them to carry them out and limit themselves to doing their chores in the way that they have been instructed.

The problem of moral distance and hierarchy is prominent in business and AI ethics, where the distance between layers of organizations appears between developers and companies. In this case, the effect of authority on people who develop algorithms continues to have the moral distance implications defended by Milgram and Bauman. The issue is that developers “work in an environment which constantly pressures them to cut costs, increase profit and deliver higher quality … Managers might coerce ICT professionals to make unethical or at least disputable decisions to the so-called benefit of the company” (Van den Bergh and Deschoolmeester 2010). Even though developers might have an established code of conduct and AI ethical guidelines, they face pressure from managers to design models that prioritize company interests (Mittelstadt 2019). There, some have argued about the threats of letting the industry write the rules for AI (and sponsor AI ethics research) and a type of “emissions” of high-tech industry as to how their profits are borne by society (Benkler 2019).

This subtype of moral distance also appears in human deference to AI decision-making. “Humans, some argue, will happily defer to the machine. Yet such blind deference is ill founded” (Zarsky 2016). There, awareness of algorithmic shortcomings becomes critical for those deploying AI. In this type of issue, “the distance de-skills us: we become dependent on the technology and we do no longer know how it works, what it does, and indeed what we are doing” (Coeckelbergh 2013).

For example, in the case of Amazon, where a data analytics program rates and fires drivers, there is no human intervention in the decision, even the dismissal notice is automatic, and there are also no prior notification protocols so that drivers can appeal the decision before their termination. The company completely defers to the decisions made by the data analytics program.

#### Complex processes

The second implication of bureaucracy and distancing emerges from *complex processes*. The latter implies the act of several persons, which we relate to the so-called problem of ‘many hands.’ Dennis Thompson (1980) was one of the first authors to state this issue. According to him, since many officials contribute in several ways to government decisions and policies, is hard to identify who is morally responsible for those policies' outcomes, creating the problem of ‘many hands.’ In situations that involve several people's performances, individuals tend to diminish the ethical implications of their acts. Hence, “loosely, this problem may be described as the problem of attributing or allocating individual responsibility in collective settings.” (van de Poel and Zwart 2015). Also, this problem of ‘many hands’ has two varieties: backward-looking (when something went wrong and the responsible is unknown) and forward-looking (when there is a need for collective action to accomplish something). There are many real examples of the two varieties as a financial crisis, global warming, or poverty, and on a small scale could be the bankruptcy of a company or the lack of communication of processes in an organization (van de Poel and Zwart 2015).

Bauman (1989) talks about the relationship of this problem to moral distance when he explains that “with most of the socially significant actions mediated by a long chain of complex causal and functional dependencies, moral dilemmas recede from sight, while the occasions for more scrutiny and conscious moral choice become increasingly rare.”

The distance created from complex situations and the problem of ‘many hands’ is a recognized problem in the field of ethics of technology (van de Poel et al. 2012; Coeckelbergh 2020). Helen Nissenbaum (1996) correlates the problem to technology and explains its implications for moral responsibility, she defends that computer systems are products of groups of individuals and organizations, and if a “system malfunctions and gives rise to harm, the task of assigning responsibility—the problem of identifying who is accountable—is exacerbated and obscured.” Also, that product finally impacts the life of an end user.

For AI this issue appears in the problem of inscrutability where “the degree to which an algorithm is inscrutable contributes to our ability to identify, judge, and correct mistakes in algorithmic decisions” (Martin 2019a). This type of algorithmic opacity, “an opacity that stems from the mismatch between mathematical optimization in high dimensionality characteristic of machine learning and the demands of human-scale reasoning and styles of semantic interpretation” (Burrell 2016) is critical to the understanding of how AI creates moral distance. The said implies that, if the logic of an algorithm is incomprehensible for those who deploy it, an insurmountable moral distance problem would appear.

For example, in the case of either the university admittance program or the Amazon program to rate and fire drivers, moral distance is created, between the manager and the individual impacted by the decision, due to a lack of understanding of how the decisions are made by the program.

#### Principlism

Finally, the third contributor to bureaucratic moral distancing is principlism. We refer to principlism as the kind of moral distance that creates a blind attachment to guidelines and principles, which sometimes ended up in unfairness. Huber and Munro (2014) first conceptualized this issue as *ethical violence* which they defined as a type of moral distance and a blind attachment to guidelines and principles, on those situations in which, by adhering to ethics, people end up interposing a distance with the specific circumstance. At first studies of moral distance described the problem as something of bureaucracy and formal rationality. However, research developed to outline the problem as something that can appear “even in informal personal relationships … and may be even implicit in the notion of ethics itself … where ethical principles can serve to justify condemnation and cruelty in the name of ethics” (Huber and Munro 2014).

In the field of AI, this type of moral distance appears in how AI guidelines, recommendations, or standards proposed for the field, may not be a solution in several scenarios. Although the use of ethical guidelines in all types of organizations has been questioned, the effectiveness of ethical codes in the field of technology has a special skepticism (Van den Bergh and Deschoolmeester 2010; see also Bia and Kalika 2007). Mittelstadt (2019) argues how *principles alone cannot guarantee ethical AI*, and how a principle-based approach “may have limited impact on design and governance.”

Taking these characteristics of AI, within the idea that principles cannot guarantee the ethics of AI, Mittelstadt defends that “going forward, AI ethics must become an ethics of AI business,” to avoid the abstraction of principlism. There “if robotics systematically enters into our interpersonal sphere, the symmetric reciprocal meeting … where the other is a unique and mortal individual (Levinas 2017a, 2017b) and elicits an ethical imperative, risks being subverted by the reduction to some quantitative computational measures which are digitally representable and match whatever criteria implemented in or feeding the program running the robot” (Nørskoy 2021). What we defend here is that the “codification of ethics,” (top-down, bottom-up, or hybrid approaches (Allen et al. 2005)) in AI may end up in a principlism moral distance problem.

Within a different realm, Haas (2020) identifies a similar issue when attempting to develop AI using reinforcement learning. Haas offers a framework to train AI models using their environment including being explicit as to the value-laden assumptions, acts, and labeling in that environment. As such, AI models learn a type of ethics given the environment developer provides for learning.

This moral distance problem could also be related to two of the unethical risks of the translations of AI principles into practices, identified by Floridi (2019). Concretely to ethical shopping and ethical washing, and how private or public institutions *shop* “for the principles that best fit its current practices, publicizes them as widely as possible and then proceeds to bluewash its technological innovation without any real improvement, much lower costs, and some potential social benefits.”

For example, in the case of learning analytics, algorithms may be designed to avoid unfairness and promote equality, and there to evaluate prospective students with the same variables. However, applying the same ratio, a principle, to students with different circumstances could be unfair to those students who do not fit the rule formalized in the program. By relying on the algorithm, developed by individuals and/or trained on historical data, to provide principles of admittance for future students, the university prioritizes those rules over those students who do not fit the mold of the majority who are well represented in the data and algorithm.

## The ethics of care as a bridge for moral distance in AI

We propose the ethics of care as a moral framework for AI ethics and the problem of moral distance. Ethics of care appeared with Carol Gilligan's and Nel Nodding’s approaches to ethics. The theory was in response to the orthodoxy of ethics of justice, since the theory is not bolstered on inviolable impartial principles but appeals to relationships and context (French and Weis 2000; see also Held 2006). Gilligan’s proposition was a response to the work of her advisor Lawrence Kohlberg.^1^Kohlberg proposed three levels of moral development and six stages (two stages within each level).^2^

However, according to Gilligan, there is an essential problem in Kohlberg’s theory since it is empirically based on a study of eighty-four boys studied for 20 years. In her pioneering study, *In a different voice*, Gilligan argued that the lack of representativeness of the original sample inhibits the claims of the universality of this theory of moral development. This is also the reason why those groups excluded from the sample hardly reach the higher stages of the sequence. What Gilligan noticed was that judgments of women appeared to exemplify the third stage of the sequence, when morality is conceived in interpersonal terms and to be good is equated with helping and pleasing the other.

Hence, the ethics of care has its roots in a feminist conversation. Gilligan's focus was on defending the positions of women, and their voices. Also, his book *In a different voice* has a subtitle *Psychological theory and women’s development*. However, Gilligan and scholars in the field of ethics of care, have long argued that she was referring to a different voice in its broader sense, not only limited to women. Hence, is essential to address this theory as a complementary and integrative approach in relation to other ethical theories such as virtue ethics or ethics of responsibility, to name a few.

The ethics of care asks us to focus on the concrete situation and provide answers concerning circumstances and context (Gilligan 1982). Hence, the ethics of care is framed as a moral vision centered on the individual. While other ethical theories such as deontology, utilitarianism, or consequentialism respond to ethical principles and duties, care ethics focuses on fostering people’s vulnerabilities and needs (Weltzien and Melé 2009). Therefore, care should be understood as a *practice and a work that must be done on a direct level* (Sander-Staudt and Hamington 2011), as a value, and as an activity. In this sense, it can be argued that the ethics of care proposes solutions according to the interests of each party and not to previously established norms (Reiter 1996).

The ethics of care has been proposed in situations where the interests of the least advantaged stakeholders are not being considered. In other words, where the distance between those making the decisions and those impacted by the decisions is too great and the marginalized stakeholder’s interests are not being seen or judged to be legitimate. The notion of care has also been proposed in the field of technology ethics. Marion Hersh (2016) suggested the ethics of care (along with narrative ethics and virtue ethics) for engineers to make conscious ethical decision-making, and for the understanding of notions of culture, diversity, and the “other”, and issues related to these. According to Hersh, the understanding of those notions is “very important for engineers for a number of reasons, including the need to design technologies, goods, and services for people who are not engineers and who are also different from them on other characteristics such as gender, race, and disability.”

Along the same line, Maurice Hamington (2019) proposed the integration of care ethics and design thinking, which is a practice from engineering (later adapted to business) to “enable innovation and problem solving through participatory processes.”

Since the essential idea of design thinking is to consider those who will use the design, “including their emotions and ambiguities,” the author proposed care ethics as a framework to help in the understanding of the end user of products and services.

The ethics of care have been applied to different areas in the business world since the 1990s (Melé 2014). There are also studies that propose ethics of care as a moral framework for stakeholder theory (Wicks et al. 1994; Burton and Dunn 1996; and Engster 2011). Burton and Dunn (1996) proposed care as a moral grounding to stakeholder theory and management decision-making, and stated a principle: “Care enough for the least advantaged stakeholders that they not be harmed; insofar as they are not harmed, privilege those stakeholders with whom you have a close relationship.” In essence, for the notion of care, firms must avoid any possible form of harm and take moral sentiments that we all share when making decisions (Wicks et al. 1994).

According to Daryl Koehn (2011), care ethics is the necessary framework in the context of unforeseen and unintended consequences because of the theory’s flexibility and reference to interdependence, empathy, sympathy, and trust, rather than rules.

Since its establishment, the notion of care has developed from an ambiguous concept to a more rigorous definition. Daniel Engster (2011) proposed a definition of care ethics as a “theory that associates moral action with meeting the needs, fostering the capabilities, and alleviating the pain and suffering of individuals in attentive, responsive, and respectful ways.” Hence, the ethics of care goes in line with avoiding harm and taking vulnerabilities, relationships, and context, as well as emotions and empathy as appropriate guides to ethical decision-making (Sander-Staudt and Hamington 2011).

Care ethics involves attention and empathic response, a commitment to attend to legitimate needs (Noddings 1984).^3^ However, the ethics of care is not altruism, or feeling sorry for someone, or making decisions to do someone a favor. Rather, “although we often think of care in terms of characteristics such as understanding, responsiveness and nurturance, the practice of care is not always and necessarily harmonious … [and] … may be imbued with conflict” (Simola 2010; see also Simola 2015).

Within the study of the ethics of care, there exists a shared understanding that ethical decision-making should consider *interdependent relationships*, *context and circumstances*, attend to particular *vulnerabilities*, and also should respond to different points of view, and hear different *voices*. We emphasize four ideas within the ethics of care that may help to ameliorate the problem of moral distance. These four ideas, although there are outlined to facilitate their understanding, would not serve if taken as a set of principles to check. These notions are presented as concepts opening a new conversation and guiding the critical thinking of those who design, develop, and deploy AI. Principles are maxims or imperatives; these notions are concepts that can’t be reduced to definitions but appeal to reflection and analysis.

### Interdependent relationships

In the ethics of care, individuals and interests are not isolated but rather have meaning in a web of interdependent relationships. Within the ethics of care, we are to not only maintain relationships but also be responsive to the needs of others. Rather than a focus on principles, the ethics of care appears as a method and a way to orientate toward the world (Sander-Staudt and Hamington 2011).

The essence of this notion is that responsibility and morality can only be understood in a network of relationships, where one puts aside the general standard and look to the concrete situation, and there “the generalized other” becomes “the particular other,” a specific individual in a particular circumstance. Also, “the ideal of care is thus an activity of relationship, of seeing and responding to need, taking care of the world by sustaining the web of connection so that no one is left alone” (Gilligan 1982). According to Nel Noddings (1984), this language “concentrates on relationships, needs, care, response, and connection rather than principles, justice, rights, and hierarchy.”

The notion of interdependent relationships differs from the idea of justice in how from a justice approach, individuals are understood as in a contest of rights, in which one talks about *transactions*. While from the notion of interdependent relationships one talks about *care*. Hence, when applying this notion to AI, it would be essential that models do not take individuals as opponents “in a contest of rights but as members of a network of relationships on whose continuation they all depend” (Gilligan 1982). Developers would be in charge of understanding interdependent relationships and how they can be impacted by algorithms. An example of the application of this notion can be illustrated in the field of learning analytics, when universities decided which students retain in a program using AI. With algorithm decision-making, universities consider quantifiable variables but ignore essential facts that could also add value to the student’s profile. For instance, a student with lower grades may have *interdependent relationships* impacting his or her scores, such as family dependents (as infants, grandparents, or parents with illness), but show commitment and responsibility. Also, this may be the case when students lower their performance because of the dedication of their time to volunteering or leadership of student associations.

### Context and circumstances

The ethics of care is a practice and something to be done on a direct level and may be understood as a “motive, ideal, virtue, and method” (Sander-Staudt and Hamington 2011). Moreover, “care theorists generally agree that care is a relational approach to morality that entails contextualized responsiveness to particular others in a manner that supersedes the mere delineation of rules or calculated consequences” (Hamington 2019). Consideration of context and circumstance would address the problem of proximity (space, time, and culture) in moral distance. Those working with AI would seek to understand the direct consequences their actions have on others, bridging the impact of a “complex process” on moral distance. Those who develop and deploy AI are responsible for ensuring that algorithms do not eliminate variables such as context (like space and time), circumstances, or historical-cultural background.

For example, within learning analytics, this notion appears as relevant when context and circumstances may affect someone’s GPA and its acceptance in a program. Students’ grades can be lower in disadvantaged neighborhoods or countries if teachers and school supplies are not optimal, but someone who comes out in unprofitable circumstances has a lot of courage, strength, and determination. Also, in the case of the Amazon firing bot, former Amazon managers explain that “the largely automated system is insufficiently attuned to the real-world challenges drivers face every day” (Soper 2021).

### Vulnerability

Within the ethics of care, understanding vulnerability is vital to understand the relevance of the needs and suffering of others and to act according to those who can be affected by a decision. Developing AI based on the ethics of care implies that algorithms do not prevent individuals from meeting their needs. When applying ethics of care to AI, those who develop and deploy AI could certify that algorithms do not prevent the possibility of fostering the needs of protected classes, people at risk of social exclusion, or marginalized stakeholders. Also, those working with AI could guarantee that the data used does not imply exploiting the vulnerabilities of those affected by the algorithm and that vulnerabilities are not used as variables to prevent their future enhancement.

For example, admittance decisions would want to address the specific *vulnerabilities* of students, such as a student with an attention deficit disorder which affects the student’s capacities in some subjects but not in others.

### Voice

When utilizing care ethics, one would identify and hear the range of voices impacted by the decision. According to Carol Gilligan (1982), “to have a voice is to be human. To have something to say is to be a person. But speaking depends on listening and being heard; it is an intensely relational act.” For Gilligan, in *In A Different Voice*, for decision-making and morality is critical to give voice to every affected part in any situation. Communication is the way to resolve ethical conflicts because when communicating, one hears different voices, opinions, and points of view. Hence, the needs of all those who are affected by an action are considered. The notion of voice essentially differs from the idea of autonomy in how voice focus is on a relational act of listening and bringing close. The idea of autonomy implies a transaction of two independent bodies where the focus is on independence and not on interconnectedness.

In the case of moral distance and AI, this notion addresses the types of proximity distance, giving voice to each culture and those far in space and time. Although AI decision-making eliminates physical interaction, moral issues created by that distancing could be addressed by listening to the voices of those affected, while those who develop AI models understand it as a priority to consider the interests of all parties.

This notion could ameliorate the problem of proximity distance if algorithms maintain open the possibility of hearing different voices and not silence any voice that should have been part of the situation in which is applied. There might be various formulas to give voice to all stakeholders, especially the most marginalized, like the work of interdisciplinary and intercultural teams working to develop and deploy AI. In the try to give voice to every affected part, those working on AI are considering the implications of the passage of time, the interests of those who are not present (whom they cannot see or who may never have a physical approach), and the needs and values according to different cultures.

For example, a university can lower the voice of marginalized groups with the application of a model which does not represent their situation or case very well. Similarly, the implementation of Amazon’s program to fire their drivers through an automated email quite directly silences the voice of the drivers who are not given a chance to contest the decision.

## Conclusions

The purpose of this paper was to identify and analyze the ethical distance created by AI in decision-making and to contribute to the proposition of ethics of care as a form to counteract and mitigate the ethical implications of AI.

The discussion about ethics, distance, and technology is essential for AI ethics within business. Firms use algorithms without specific knowledge of their procedures, such as the COMPAS algorithm used in court sentencing (Martin 2019b). There the “exclusivity of the individual is lost for the sake of technological palatability and optimization”.

(Nørskoy 2021). The distance and technologies de-skill firms’ employees and make it easier to make decisions that could change a person's life, like decisions on an employee termination or acceptance to a university.

We examined how the abandonment of decision-making to AI has the ethical implication of moral distance. There we explained how moral distance arises from a proximity distance (of space, time, and culture) and from a bureaucratic distance (in the form of hierarchy, within complex processes, and principlism). We stated how these types of moral distance are presented in how AI works. Moreover, we argued that AI exacerbates the problem of moral distance. Finally, we have proposed the ethics of care as a moral framework and four concepts within this moral theory that can help contextualize the other and make *closer* the person who is at distance. These four ideas, *interdependent relationships*, *context and circumstances*, *vulnerability*, and *voice* would not serve if taken as a set of principles to check. Principles are maxims, sometimes presented as dogmas, which do not allow reflection. These notions are concepts opening a new conversation for the critical examination of AI.

Similar to all other ethical theories, the ethics of care can be considered unrealistic or not able to stop all moral problems or harms (Hamington 2019). We are arguing that the ethics of care is useful to analyze algorithmic decision-making given AI’s negative impact on moral distancing. In this way, the ethics of care is useful given this particular context. In addition, the application of the ethics of care is to aid in the use of AI. The goal is to offer a mechanism to design and develop algorithmic decision-making tools that take into consideration the ethics of care.

Future research should study the differences, and specific implications of the legal, moral, epistemic, and practical aspects of distancing. Also, future research should address the whole range of feminist theory, and how hearing this different voice in the technology sector may ameliorate the problem of moral distance given the problem of representativeness in the workforce.

## Footnotes

1 L. Kohlberg, *The development of modes of thinking and choices in year 10 to* 16 (Ph.D. Diss., University of Chicago, 1958). L. Kohlberg, *Essays on Moral Development* (Harper & Row, New York, 1981).

2 The first was the *Preconventional level*, with stage 1. The punishment-and-obedience orientation, and stage 2. The instrumental-relativist orientation. The second one was the *Conventional level*, with stage 3. The interpersonal concordance “good boy—nice girl”, and stage 4. The “law and order” orientation. Finally, the third level was the *Postconventional, autonomous, or principled level*. In this last level was the stage 5. The social contract, legalistic orientation, generally with utilitarian overtones, and stage 6. The universal ethical-principle orientation. See L. Kohlberg and R. H. Hersh, *Moral Development: A review of the theory*, “Theory Into Practice” 16/2 (1977) 53–59.

3 Also, “care” should be distinguished from “personal service,” the former “involves meeting the needs of those who are unable to meet such needs themselves, the latter involves meeting needs for others who could meet such needs themselves.” (Sander-Staudt and Hamington 2011; see also Noddings 1984).
